# Cannabis use, abuse and dependence during the COVID-19 pandemic: a scoping review

**DOI:** 10.1007/s00702-022-02564-8

**Published:** 2022-11-08

**Authors:** Udo Bonnet, Michael Specka, Patrik Roser, Norbert Scherbaum

**Affiliations:** 1 Department of Psychiatry, Psychotherapy and Psychosomatic Medicine, Evangelisches Krankenhaus Castrop-Rauxel, Academic Teaching Hospital, University of Duisburg-Essen, Grutholzallee 21, 44577 Castrop-Rauxel, Germany; 2 Department of Psychiatry and Psychotherapy, Faculty of Medicine, LVR-Hospital Essen, University of Duisburg-Essen, Essen, Germany

**Keywords:** Cannabis addiction, Cannabis use disorder, SARS-COV-2, COVID-19, Hospitalization, Mortality

## Abstract

The interaction between cannabis use or addiction and SARS-COV-2 infection rates and COVID-19 outcomes is obscure. As of 08/01/2022 among 57 evaluated epidemiological/clinical studies found in Pubmed-database, most evidence for how cannabis use patterns were influenced by the pandemic was given by two systematic reviews and 17 prospective studies, mostly involving adolescents. In this age group, cannabis use patterns have not changed markedly. For adults, several cross-sectional studies reported mixed results with cannabis use having increased, decreased or remained unchanged. Two cross-sectional studies demonstrated that the severity of adults´ cannabis dependence was either increased as a consequence of increasing cannabis use during the pandemic or not changed. Regarding the effect of cannabis use on COVID-19 outcomes, we found only five retrospective/cross-sectional studies. Accordingly, (i) cannabis use did not impact mild COVID-19 symptoms; (ii) cannabis using individuals experienced more COVID-19-related hospitalizations; (iii) cannabis using veterans were associated with reduced SARS-COV-2 infection rates; (iv) frequent cannabis use was significantly associated with COVID-19 mortality, and (v) cannabis dependents were at higher risk of COVID-19 breakthrough after vaccination. It should be outlined that the validity of these retrospective/cross-sectional studies (all self-reports or register/e-health-records) is rather low. Future prospective studies on the effects of cannabis use on SARS-COV-2 infection rates and COVID-19 outcomes are clearly required for conclusive risk–benefit assessments of the role of cannabis on users’ health during the pandemic. Moreover, substance dependence (including cannabis) is associated with (often untreated) somatic comorbidity, which severity is a proven key risk factor for worse COVID-19 outcomes.

## Introduction

Since late 2019, SARS-COV-2 is plaguing the world. Meanwhile, we have experienced three infection waves with different leading SARS-COV-2 variants and decreasing serious morbidity and mortality quotas, especially due to social lockdowns and vaccination strategies (El-Shabasy et al. 2022). Yet, a fourth wave seems to arise in some Western countries. Risk factors for serious COVID-19 disease and related mortality are currently under intense investigation. The same applied to protective factors beyond social lockdown and vaccination. In this context, the role of cannabis use is a matter of the actual debate. Does it help against COVID-19 due to favourable immunomodulatory features of some of its ingredients in cells? (Paland et al. 2021; van Breemen et al. 2022). Or does it increase COVID-19 morbidity and mortality? In this context, bio-social factors such as possible increased combustive (Rosoff et al. 2021) cannabis use or cannabis use-related behavioural non-adherence to prevention recommendations for COVID-19 mitigation or vaccination (Monnig et al. 2021) are discussed. At this juncture, an association between heavy drinking and non-adherence to COVID-19 public health guidelines have already become more prevalent (Fendrich et al. 2021). Moreover, social distancing guidelines and sequential stay-at-home orders caused the partial closing of the majority of outpatient substance use clinics, addiction counselling facilities as well as social services. This might also have worsened the health situation of cannabis dependents, thereby possibly increasing their vulnerability for severe COVID-19 symptoms.

This scoping review aims to shed more light on potential changes of cannabis use and dependence during the pandemic, also considering current as well as COVID-19-related health conditions of cannabis using people.

## Methods

We performed a scoping review (Peters et al. 2015) of the relevant literature using the search term combinations “cannabis” AND “marijuana” AND “COVID-19” OR “SARS-COV” in the Pubmed database (https://pubmed.ncbi.nlm.nih.gov/). As of 08/01/2022, we identified 310 records OR 57 (in sum 367) records. After subtracting doublets, we assessed 312 eligible articles which were evaluated according to clinical and epidemiological studies (57 articles). Exclusion criteria comprised all non-clinical, non-epidemiological and pre-clinical studies (in vivo and in vitro, *n* = 254) as well as clinical studies exploring the treatment of SARS-COV-2 or COVID-19 with cannabinoids (*n* = 1, Crippa et al. 2021). The results were structured according to the hierarchy of the Evidence-Based-Medicine guideline (EBM; The Oxford Levels of Evidence 2”. https://www.cebm.ox.ac.uk/resources/levels-of-evidence/ocebm-levels-of-evidence—The Levels of Evidence, version 2.1).

### Results

Meta-analyses, randomized-controlled studies or prospective representative population level surveys addressing the effects of the pandemic on cannabis use (disorder; DSM-5), abuse or dependence (ICD-10) were not found. The same applied to well controlled studies about the effects of cannabis use (disorder), abuse or dependence on SARS-COV-2 infection rates or COVID-19 morbidity/mortality. Table 1 shows the systematic reviews (EBM Level 2) and prospective studies (EBM Level 3) which were detected.

#### Systematic reviews

Two systematic reviews, both including international epidemiological studies exclusively on youths and adolescents, provided limited evidence for an increase of cannabis use during the pandemic. The smaller systematic review by Jones et al. (2021) constituted higher rates of anxiety, depression, and distress due to the pandemic. Furthermore, the results suggested that adolescents had an increased alcohol and cannabis use during the pandemic in terms of a coping strategy. These results, however, were not corroborated by a larger systematic review by Layman et al. (2022) including 20 studies with cannabis users of whom only four studies showed an increased cannabis use during the pandemic (Table 1).

**Table 1:** Systematic reviews and prospective studies considering cannabis use data during the pandemic^a^

Study/country	Measurement points/SARS-COV-2 wave	Population	Cannabis	Further results
*Systematic review*				
Jones et al. (2021) USA	First wave	Included were 16 international studies (web based self-reports) of substance use in youth/adolescents	Globally, adolescents of varying backgrounds (including LGBTQ^b^) experience higher rates of anxiety, depression, and stress due to the pandemic. Adolescents also have a higher frequency of using alcohol and cannabis during the pandemic for coping the pandemic	
Layman et al. (2022) USA	First and second wave	Included 49 international studies of substance use in adolescents/youths, relying on web based self-reports	20 studies included measures on use of cannabis, including marijuana, hashish, and edibles. Four, five, and three studies reported an increase, decrease, and no change in cannabis use, respectively. Eight studies reported neither an increase nor a decrease	Prevalence of youth/adolescent substance use has largely declined during the pandemic
*Prospective studies*				
Graupensperger et al. (2021) Washington State, USA	Two self-report surveys: prior to the COVID-19 pandemic (January 2020) and again during the initial acute phase of the pandemic (April/May of 2020) First wave	Community sample of young adults (*N* = 572; mean age = 25.1 years; 60.8% female)	No changes in cannabis use; significant use motive changes: use against boredom increased, celebration motives decreased	Increased alcohol use frequency, decreased amount per drinking occasion; significant use motive changes: increase for depression coping, decreases for social, enhancement, and conformity motives
Hawke et al. (2021) Ontario, Canada	Four self-report time points, i.e., every two months beginning in the early stages of the pandemic in April 2020 First and second wave	*N* = 619 youths/adolescents/young adults aged 14–28 years, 62.7% female, 61.4% Caucasian), urban hospital sample	No change	No change in various substance use
Pelham et al. (2021) 21 study sites across the USA	Three assessments between May and August 2020—compared to pre-pandemic surveys of the same participants (2018–2020), self-reports First Wave	The Adolescent Brain Cognitive Development Study. *N* = 7,842 youths; range = 10.5–14.6)	No change in rare cannabis use	Compared to before the pandemic, fewer youth were using alcohol and more youth were using nicotine or misusing prescription drugs
Wang et al. (2021) Florida, USA	Baseline survey between 2018 and 2020 and a brief phone survey between May and October 2020, self-report First Wave	*N* = 222 people living with HIV (mean age = 50.2, 50.9% female, 14.5% Hispanic, 64.7% Black, 15.8% White, 5% other	*N* = 122(55%) reported no change. *N* = 36 (16.2%) reported decreased use, *N* = 64/222(28.8%) described increased marijuana use, Increased use was associated with PTSD at baseline, and worsened health during the pandemic	
Chaffee et al. (2021) Northern California, USA	6 month follow up, 2019- 2020, before and after the stay-at-home order, self-report First Wave	*N* = 1006 adolescents, 62% female, 49% were non-Hispanic White; ninth- and tenth-grade students enrolled at 8 public high schools	No relevant change	No relevant change of tobacco and alcohol use, decline of physical activity
Leatherdale et al. (2021) Quebec/Ontario, Canada	Online; early stages of the COVID-19 pandemic period, 2020, self-report First wave	*N* = 1937 cannabis using schooled youths	No relevant change	
Cousijn et al. (2021) The Netherlands	On-line between January 2019 and May 2020, self-report First Wave	Daily cannabis users (*N* = 120, aged 18–46) vs Controls (*N* = 63, aged 18–31)	Significantly increased cannabis use during lockdown, but *no increase in cannabis use disorder symptom severity* and mental health problems. Cannabis users experienced 30% job loss and significantly increased loneliness, while contact with partners and families significantly improved	
Rogés et al. (2021) Central Catalonia, Spain	Before lockdown (October 2019–February 2020) and 2 months after the lockdown ended, self-reports First Wave	*N* = .303 schooled adolescents aged 14–18	Trend of reduction of cannabis use with the exception of Vocational and Educational Training (VET)-students (increase of hazardous cannabis use)	Trend of reduction of consumption with the exception of Vocational and Educational Training (VET)-students (increase in binge drinking, hazardous drinking of alcohol, and daily smoking of tobacco
Dumas et al. (2022) Ontario, Canada	Four self-report internet surveys (spring 2020),starting during the first stay-at-home order and ending approximately 14 months later First and second wave	Adolescents (*N* = 1068, 14–18 years, mean age = 16.9 years and 76.7% female)	Significant decreases and increases in cannabis use during the first stay-at-home and re-opening orders, respectively, but not during the second lockdown and re-opening	The same applied to alcohol use and binge drinking
Otiashvili et al. (2022) Tsibili, Georgia	Bi-weekly online survey in April-September, 2020, self-reports First Wave	50 drug using adults (mean age: 36; 22% female), recruited through a snow-ball sampling	Use of cannabis products declined significantly	Participants used significantly fewer substances, including alcohol and diverted methadone/buprenorphine
Pocuca et al. (2022) Quebec, Canada	Quebec longitudinal study of child development, who completed prepandemic (2019; 21 years) and COVID-19 (mid-March to mid-June 2020) surveys, self-reports First Wave	*N* = 1,080; 54% female) young adults, 21–22 years	No changes in cannabis use	Significantly decreased binge drinking, no changes in alcohol use
Meanley et al. (2022) USA	Two pre-COVID-19 (October 2018–September 2019) and one COVID-19-era (April 2020–September 2020), time-points within the MACS/WIHS Combined Cohort Study (MWCCS), self-reports First Wave	People living with HIV (*N* = 2121, 53.5 years (SD = 10.1)), predominantly people of color (73.6%), 62.3% females,	No change of cannabis use	Binge drinking and recreational drug use decreased at the beginning of the pandemic. male sex and having depressive symptoms early pandemic were positively associated with each substance use outcomes. Social support was negatively associated with recreational drug use
Patrick et al. (2022) USA, National Sample	Followed from the 12th grade in spring 2019 to fall 2020, self-reports First wave	*N* = 1244, mean age 19.6 years	No information about change; COVID-19-related isolation was associated with marijuana use	8.9% increased vaping, and 8.2% increased drinking to cope with social distancing and isolation
Imtiaz et al. (2022) Canada	Repeated cross-sectional design; data from six waves of a national, online survey of adults residing in Canada who spoke English (May-08 2020 to December-01 2020) First wave	*N* = 6,021; 18–> 50 years	No change	
Sznitman (2022) Israel	Two online surveys corresponding to the first and the second lockdown periods First and second wave	*N* = 116 monthly adult cannabis users	Increased cannabis use, solitary use, and use before noon during both lockdown periods. Lockdown coping motives were related to reported increases in cannabis use. People with increased cannabis use at lockdown two reported *more dependence symptoms*	
Dietz et al. (2022) Mainz, Germany	Three online surveys, pre-pandemic 2019 during pandemic, 2020, during pandemic 2021 First and second wave	University students First survey: *N* = 4350, mean age 23.8; second survey *N* = 3066, mean age = 23.4; third survey *N* = 1438, mean age = 23.7	Decreased cannabis use for neuroenhancement	Decreased pharmacological neuroenhancement per se
Sylvestre et al. (2022) Montreal, Canada	Ongoing longitudinal study (since 1999, including at that time 12–13 years old youths of 10 high schools); Pre-pandemic data on use of combustible cigarette, e-cigarette, alcohol, binge drinking and cannabis were collected at ages 20.4, 24.0 and 30.6. Data were again collected from December 2020 to June 2021 Second wave	Nicotine Dependence in Teens study, Montreal, Canada, 2007–21 *N* = 704 young adults, mean age: age 33.6, 60.2% female	Compared to the beginning of the study significantly decreased weekly/daily cannabis use (2007: 44.3%, 2021; 23%). The last pre-pandemic investigation revealed 17.5% -indicating an increase during the pandemic Compared to the beginning of the study significantly decreased weekly/daily use of alcohol (90.6% vs 44.2%), binge drinking (76% vs 7.9%), combustible cigarettes (47.1% vs 11.9%, e-cigarettes (NA vs 5.4%). The last pre-pandemic investigation revealed for alcohol 49.1%, for binge drinking 9.1%, combustible cigarettes 18%, e-cigarettes 5.4%—indicating a slight decrease of alcohol use, binge drinking and cigarettes use as well as a slight increase of the e-cigarettes use during the pandemic Low education level and living alone were associated with higher risks of initiated/increased use of most substances	

^a^Published in PubMed up to 08/01/2022

^b^Lesbian, gay, bisexual, transgender, and queer (LGBTQ) adolescents

#### Prospective studies

We identified 14 prospective studies and three “quasi-prospective” studies (using a repeated cross-sectional design, Imtiaz et al. 2022; Dietz et al. 2022; Sylvestre et al. 2022). Most prospective studies were conducted with young adults, adolescents and youths from USA, Canada and Western European Countries which described no change of cannabis use during the pandemic (Table 1). One study explicitly described significant decreases and increases in cannabis use during the first stay-at-home and re-opening orders, respectively, but not during the second lockdown and re-opening (Dumas et al. 2022). Another study did not find any change of cannabis use but points to a link of adolescent cannabis use with the uncomfortable lockdown conditions (Patrick et al. 2022).

Still four of the 17 prospective studies shown in Table 1 described a non-fluctuating increase of cannabis use. A Spanish study in schooled adolescents found a small subgroup of Vocational and Educational Training (VET) students who showed an increase of risky cannabis use, together with an increase in binge drinking, hazardous drinking of alcohol and daily smoking of tobacco (Rogés et al. 2021). The remaining three studies reported an increase of cannabis use among adults. An Israeli study found an increase of solitary use in pre-pandemic monthly cannabis consumers which was related to an increased incidence of dependence (Sznitman 2022). A Dutch study also described an increase of cannabis use, but no increase of cannabis use disorder or other mental health problems (Cousijn et al. 2021). Within a cohort of 222 adult people living with HIV (PLWH), Wang et al. (2021) identified a subgroup of 64 mid-fifties (28.8%) who confirmed an increase of their cannabis use which led to a worsening of their general health condition or to the reactivation of a pre-existing post-traumatic stress disorder during their lockdown experience (Wang et al. 2021).

The remaining PLWH-population investigated by Wang et al. (2021) reported either no change (55%) or a decrease (16.2%) of their cannabis use (Table 1). A further US-study on cannabis-using PLWH’s reported no change of their cannabis consumption during the pandemic (Meanley et al. 2022).

All studies summarized in Table 1 had an epidemiological approach and almost all of them relied on self-reports, were conducted online and comprised the period of the first and, to a smaller amount, also the period of the second SARS-COV-2 infection wave. We did not find any prospective study about the impact of cannabis use (disorder), abuse or dependence on the course or outcome of SARS-COV-2 infection or COVID-19.

#### Wastewater-based epidemiology

In Portugal and Spain, no significant changes of alcohol, tobacco, cannabis and opioid concentrations were found in population-normalized loads in 2020 compared to former years (Estévez-Danta et al. 2022). In Reykjavik, cannabis use seemed to be stable from 2017 to 2019, thereafter showing signs of an increase during the pandemic in 2020, while there was a continuous increase of amphetamine and methamphetamine signals since 2017 and a decrease of cocaine signals in 2020 (Löve et al. 2022).

## Other studies

### Sales activity

Increases in sales activity related to cannabis during the first lockdown were detected on the darknet (Groshkova et al. 2020) as well as the legal US markets (Schauer et al. 2021).

### Post-mortem epidemiology

A Finnish study re-evaluated all post-mortem toxicology cases at least positive for buprenorphine, amphetamine or cannabis. The number of monthly cases within the first 8 months of the year 2020 was compared with the number of baseline cases from 2015 to 2019. Following the stay-at-home orders in March 2020, the numbers of buprenorphine, amphetamine and cannabis findings increased (Mariottini et al. 2020).

### Cannabis use

#### Changes in various populations

We detected numerous epidemiologic cross-sectional and retrospective questionnaire studies from various countries and different populations showing mixed results for cannabis consumption trends during the pandemic, with some of them showing an (occasionally marginally) increased cannabis use (e.g., Vanderbruggen et al. 2020; Manthey et al. 2021; Assaf et al. 2022) and others which reported either a decreased cannabis use or no change (e.g., Nguyen et al. 2021; Scherbaum et al. 2021; Manthey et al. 2021). Participants who experienced two lockdowns reported more frequent consumption of alcohol and cannabis in the past 30 days than those who experienced only one lockdown. After adjustment for demographic variables, significant differences in the consumption of alcohol were found between participants who experienced one lockdown compared to those who experienced two lockdowns, whereas there were no differences regarding the use of cannabis (Bonny-Noach et al. 2021).

#### Own investigation during the early first SARS-COV-2 wave

In a multi-center cross-sectional survey conducted 2021, we investigated adult patients (*N* = 332, 23% females, median age 40 years, 44% with migrant background, 73% unemployed, 49% in a stable relationship, 13% living with children) admitted to in-patient drug detoxification treatment. A general increase of their cannabis use due to the COVID pandemic was reported by 7.2% of participants, a decrease by 6.3%.

The rate of self-reported current cannabis consumers (last 30 days before admission to treatment) was 40.3%, and 0.3% had recently used synthetic cannabis. This can be compared with a previous multicentre study carried out 2018/2019 in the same region (Specka et al. 2020) and with a similar patient sample (*n* = 295, 79% male, median age 39 years, 42% migrant background, 46% in a stable relationship, 13% living with children, 74% unemployed). There, 53% of patients had reported recent cannabis use, and 2% recent use of synthetic cannabis.

Among those patients with a cannabis use disorder (CUD) in the 2021 survey (*n* = 113), the majority (*N* = 65, 65.7% of *n* = 99 with valid answers) reported no pandemic-related change of their cannabis use; 16 patients (16.2%) reported an increase and seven (7.1%) a decrease. Eleven participants with a CUD (11.1%) reported a fluctuating pattern of increased and decreased use. In addition, of those CUD patients with lifetime use of synthetic cannabinoids (*n* = 20), none reported an increased use of that substance class, and two reported a decrease due to the pandemic.

#### Medical cannabis

Medical cannabis is usually prescribed for chronic health conditions. Therefore, most medical cannabis users are assumed to be at an increased risk of serious COVID-19 morbidity and mortality. Per internet survey conducted in Florida, adult medical cannabis users (*N* = 1202) were evaluated with regard to the alterations of their usual cannabis use practice and their health situation from March to April 2020. Mental health (76%), chronic pain (44%), cardiometabolic (33%), respiratory (17%) and autoimmune conditions (12%) were mostly reported as indications for the prescription of medical cannabis. More than half of them reported being afraid of COVID-19 and transferring the virus to someone else. Two percent (2.1%) had been tested for SARS-COV-2 infection with only one positive test result. Respondents with mental health problems described increased medical cannabis use by 91% since COVID-19 was declared a pandemic compared to those without mental health problems. A minority (16%) had switched their route of medical cannabis administration from smoking to non-smoking forms (Vidot et al. 2021).

Medical and recreational cannabis use by cancer patients were found not to be markedly influenced by the pandemic-related restrictions according to a small study from Florida within the first SARS-COV-2 infection wave (Donovan and Portman 2021). Another US study, however, reported a “substitution” of the limited access to medical cannabis by increasing recreational cannabis use or use of non-cannabis drugs, mostly alcohol and sleeping pills (Boehnke et al. 2021).

#### Cannabis use and COVID-19 public health guidelines

Cannabis as well as cigarettes, electronic cigarettes or stimulant use were not significantly associated with behavioral adherence to Centers for Disease Control and Prevention Guidelines for COVID-19 Mitigation (USA), after adjustment for sociodemographic variables and other substance use covariates (Monnig et al. 2021), in contrast to problematic alcohol use (Fendrich et al. 2021).

#### Cannabis use and mental health

Loneliness and boredom, stress, reactive anxiety and depression as well as social distancing were identified as driving factors for cannabis use, as well as for intake of alcohol, tobacco and other drugs in adolescents and adult populations during the pandemic (e.g., Fitzke et al. 2021; Somé et al. 2022; Reilly et al. 2022; Gutkind et al. 2022). In adolescents, cannabis use was related to larger functional impairment in daily activities and this relation was mediated by the sequential effects of difficulty with emotion regulation and pandemic-related distress. The same applied to alcohol and other psychoactive drugs including analgesics (Jones et al. 2021; Ismael et al. 2021; Kumar et al. 2022).

##### Cannabis use disorder, cannabis abuse and dependence

An increase of suspected cannabis hyperemesis syndrome (CHS; Bonnet 2022) in adolescents during the first SARS-COV-2 wave in Baltimore and St. Petersburg, USA was constituted (Lonsdale et al. 2022). In Theran, a 26-year-old cannabis-dependent male was diagnosed with CHS, which was initially confused with gastrointestinal COVID-19 (Pirnia et al. 2020). Another case report described the development of manic symptoms (Bonnet et al. 2010) resulting from more intense cannabis use to self-manage mild COVID-19 (Kaggwa et al. 2021).

In Spain, for the majority of individuals with substance use disorder (SUD), their substance use remained stable during lockdown in comparison to pre-pandemic conditions. However, in a small subgroup of SUD-patients a reduction was found in the use of cannabis, but also of tobacco, alcohol, and cocaine. Nearly 50% of the SUD population reported a deterioration in depression and anxiety symptoms during lockdown which was associated with the following risk factors: previous trauma exposure, female gender, distress and isolation, income reduction and alcohol use, but not with cannabis abuse or dependence (Blithikioti et al. 2021).

#### Cannabis use and COVID-19-outcomes

We found five studies on this issue. In a retrospective cohort study, SARS-COV-2 positive Brazilians (*N* = 993) quarantined at home during the first wave were monitored by phone for COVID-19 symptoms. All were classified to experience mild COVID-19. The number of COVID-19 symptoms was not associated with pre- or post-infection substance use, comprising cannabis, alcohol, tobacco, benzodiazepines and analgesics. Non-medical benzodiazepine use was found to have positive and negative bi-directional associations with non-medical cannabis and non-medical analgesic use, respectively (Ismael et al. 2021). The second study analyzed the electronic health records of US veterans (*N* = 5,556,315) in 2020 for SARS-COV-2 infection, SUD and specific SUDs: tobacco (13,5%), alcohol (6%) cannabis (CUD; 1.9%), opioids (1.1%), cocaine (0.9%), stimulants (0.6%), sedatives (0.2%). One percent (1.1%) of the veterans was tested positive for SARS-COV-2 infection. Among them, 19% were hospitalized, 8% admitted to ICU, and 6% died. All SUDs except for CUD was associated with testing positive for SARS-COV-2 infection, also after adjustment for demographic variables and psychiatric comorbidity. Any SUD and all substance-specific SUDs were associated with hospitalization. No SUD was associated with COVID-19 related hospitalization and mortality. Veterans treated in 2019 for SUD had significantly lower COVID-19-related mortality rates than those without SUD treatment (Hasin et al. 2022).

According to a large retrospective US-cohort study on patients diagnosed with COVID-19 (*n* = 6291 e-health records), those with a SUD (including cannabis use disorder/dependence) were associated with a greater number of hospital admissions in 2020 (Ramakrishnan et al. 2022). ICU-admissions were related to alcohol use disorder and ventilator support was associated with both alcohol use disorder and opioid use disorder, but not with cannabis use disorder (Ramakrishnan et al. 2022).

In a population-based US cohort study, the risk, time trends, outcomes and disparities of COVID-19 breakthrough infection in fully vaccinated SUD patients starting 14 days after completion of vaccination were evaluated. The electronic health records of 579,372 individuals (30,183 with SUD and 549,189 without SUD) who were fully vaccinated between December 2020 and August 2021 and had not contracted COVID-19 infection prior to vaccination, were evaluated. Among SUD patients, the risk for breakthrough infection ranged from 6.8% for tobacco use disorder to 7.8% for CUD; all these rates of SUD patients were significantly higher compared to the 3.6% in the non-SUD population. Breakthrough infection risk remained significantly higher after adjustment for demographics and vaccine types for all SUD subtypes, except for tobacco use disorder, and was highest for cocaine and cannabis use disorders (hazard ratio, HR = 2.06, 95% CI: 1.30–3.25 for cocaine; HR = 1.92, 95% CI: 1.39–2.66 for cannabis). After matching SUD and non-SUD patients for lifetime comorbidities and adverse socioeconomic determinants of health, the risk for breakthrough infection no longer differed between these populations, except for patients with CUD who remained at increased risk (HR = 1.55, 95% CI: 1.22–1.99). The risk for breakthrough infection was greater in SUD patients who were immunized by the Pfizer compared to the Moderna vaccine. The risk for death was 1.7% and 0.5%, respectively. No significant age, gender and ethnic disparities for breakthrough infection was uncovered (Wang et al. 2022).

A cross-sectional study based on the UK Biobank dataset identified 13,099 persons with cannabis smoking history. Cannabis users who smoked more than once per month were calculated to have a significantly poorer COVID-19-related survival, even after adjusting for known risk factors, such as age, gender, history of combustible smoking and comorbidity. The frequency of cannabis use was suggested as candidate predictor for mortality risk of COVID-19 (Huang et al. 2022).

### Anomalies in the context of the legalization of recreational cannabis use

Unintentional cannabis exposures in children (6 months to 5 years old) reported to United States poison centers increased significantly after the initial COVID-19 stay-at-home orders. This trend might be associated with COVID-19 quarantines, increased time children spent at home, increased availability of cannabis products in homes, etc. (Laudone et al. 2022).

## Discussion

To date, the best evidence for the situation of cannabis use during the pandemic relies on two systematic reviews and seventeen prospective studies, mostly involving adolescents. Accordingly, cannabis use seemed to have not changed apparently in adolescents (Table 1); the most relevant population at risk for the development of cannabis use disorder/dependence and related comorbidities (Hall and Degenhardt 2014; Solymosi and Köfalvi 2017). Also, the majority of a lot of cross sectional studies on various adult populations, including an own one with polysubstance-using adults, reported no relevant change of cannabis use in the most cases (e.g., Nguyen et al. 2021; Scherbaum et al. 2021; Manthey et al. 2021). The limited number of investigations using waste-water (Löve et al. 2022), post-mortem (Mariottini et al. 2020) or sales-activity epidemiology (Groshkova et al. 2020; Schauer et al. 2021), however, cast some doubt on whether there was not more hidden cannabis use as admitted within self-report questionnaires usually used throughout the pandemic research on addictive substance use (e.g., Table 1). Increasing cases of the cannabis hyperemesis syndrome (Lonsdale et al. 2022) which might also be considered as an indirect indicator of an increased cannabis use supports these concerns. If cannabis use would have increased during the pandemic, would this trend impact the health and the COVID-19 outcomes of the users? A Dutch study reported no deterioration of cannabis dependence and mental health in this situation (Cousijn et al. 2021). Contrarily, an Israeli study reported an increase of dependence symptoms (Sznitman 2022) and adolescents were observed to show larger functional impairment in daily activity along with increased cannabis use during the pandemic (Jones et al. 2021; Ismael et al. 2021; Kumar et al. 2022).

The interaction between cannabis use and individual health is multi-complex, per se considering the involvement of usually co-used alcohol, tobacco and other psychoactive drugs, comorbidity [in case of the pandemic, e.g. reactive anxiety and depression (e.g., Bartel et al. 2020; Jones et al. 2021)], as well as behavioural (lifestyle), demographic (e.g., socioeconomic status) and developmental (e.g., education, childhood trauma) risk factors and genetic vulnerability. This leads us to expect a large heterogeneity of results arising along with further research about cannabis use and people´s health during the pandemic. The results from studies up to now provide preliminary snapshots of this assumed heterogeneity, particularly limited to the first and the second waves of the pandemic.

Only a few studies have analyzed the effect of cannabis use or dependence on COVID-19 outcomes. Of these, one Brazilian phone-interview study reported no association of cannabis use and the number of mild COVID-19 symptoms (Ismael et al. 2021). Another large study on US study on veterans´ electronic health records described no negative effect of SUD including CUD on COVID-19 mortality, but demonstrated a significant relation between CUD and hospitalization rate due to COVID-19 (just as with other SUDs, Hasin et al. 2022). As far as we know, this was the first epidemiological study which linked cannabis use to the likelihood of SARS-COV-2 infections, which seemed to be reduced in comparison with other SUDs (Hasin et al. 2022). The significance of this finding for the burgeoning pre-clinical cannabinoid research on possible curbing effects on SARS-COV-2 and COVID-19 mitigation (Paland et al. 2021, van Breemen et al. 2022) is virtually impossible to assess so far without further replicating results. On the other hand, frequent cannabis use was significantly associated with COVID-19 mortality (Huang et al. 2022, UK Biobank register), and CUD patients were found to be at greater risk of COVID-19-breakthrough after vaccination in comparison with patients with other SUDs (Wang et al. 2022, e-health record study). In this context, it should be outlined that the validity of studies using registers or e-health records, although including large samples, is generally low without a prospective design. Future prospective studies on the effects of cannabis use/dependence on SARS-COV-2 infection rates and COVID-19 outcomes are definitely essential for conclusive risk–benefit assessments of the role of cannabis on the users´ health during the pandemic.

Certainly, substance dependence is associated with (often untreated) somatic comorbidity, such as cardiovascular diseases, diabetes mellitus, COPD, renal diseases, cancer and severe mental disorders (Farhoudian et al. 2020; Balaram et al. 2021), and the severity of these associated comorbidity is yet regarded as the primary risk factor for worse/fatal outcomes of COVID-19 (Farhoudian et al. 2020; Hoertel et al. 2022) but also of other infectious epidemic/pandemic diseases, such as influenza or AIDS. Further investigating the immune/resilience-modulatory role of addictive substances, such as opioids and cannabis/cannabinoids (Friedman et al. 2006, Malinowska et al. 2021, Paland et al. 2021, van Breemen et al. 2022), and non-addictive substances, such as antidepressants (Bonnet and Juckel 2022) and natural antioxidants (Choe et al. 2022), appears to be a promising challenge to find new therapeutic or preventive approaches for improving the prognosis of COVID-19 and Long-COVID.
